# Naming the menagerie: creativity, culture and consequences in the formation of scientific names^†^

**DOI:** 10.1098/rspb.2023.1970

**Published:** 2023-11-01

**Authors:** Stephen B. Heard, Julia J. Mlynarek

**Affiliations:** ^1^ Department of Biology, University of New Brunswick, Fredericton, New Brunswick, Canada E3B 6E1; ^2^ Division Collection et recherche, Insectarium de Montreal, Quebec, Canada

**Keywords:** scientific names, Latin names, nomenclature, etymology, taxonomy, species discovery

## Abstract

The coining of scientific names for newly described species is one of the most creative acts in science. We briefly review the history of species naming, with an emphasis on constraints and freedoms in the choice of new names and how they came to be. We then consider patterns in etymologies and linguistic origins of scientific names across clades and through time. Use of ‘non-classical’ languages (those other than Latin and Greek) in naming species has increased, as has the use of eponymous names (despite recent controversy around the practice). Finally, we consider ways in which creativity in naming has consequences for the conduct and outcome of scientific work. For example, sale of naming rights has funded research and conservation, while naming species after celebrities has increased media attention to the science of species discovery. Other consequences of naming are more surprising, including a strong effect of species-name etymology on the kinds of scientific studies conducted for plant-feeding arthropods. Scientific naming is a clear example of how science and scientists are socially situated, and how culturally influenced decisions such as what to name a new species can affect both public perception of science and the conduct of science itself.

## 1. Introduction

There is a persistent trope, at least in the public imagination, that creativity and the scientific enterprise are opposites—or at least that they share little common ground. As most scientists would easily recognize, this is not true. Many aspects of science involve considerable creativity: experimental design, for example, or (perhaps to the surprise of some) scientific writing [1,2]. But what is the *most* creative act in science? While that is not an answerable question, we would argue that a strong contender lies in scientific naming: the practice of coining a so-called Latin binomial, or scientific name, to apply to a newly described species. (‘Binominal’ and ‘binomial’ are synonymous; we use the latter because it will be more familiar to most readers.) We review the history of this creative act, with a particular emphasis on how and why it became as creative as it is, and we examine some of the consequences of this creativity for taxonomy and for the natural sciences that depend on it. While creativity in naming is fascinating in and of itself, its consequences matter: for science outreach, for conservation biology and even for the distribution of scientific effort across taxa.

## 2. Brief history of nomenclature and its creativity

Our current system of scientific nomenclature is to a large extent a Linnaean one; but attempts to list and name organisms have (of course) a much deeper history. The Uru.an.na, for example, is a set of Babylonian clay tablets from the seventh century BCE listing and naming medicinal plant species [3]; the Shen Nong Ben Cao Jing, or Divine Farmer's Materia Medica, is a Chinese text from *ca* AD 250 (but probably codifying much older knowledge) that lists 365 plants [4]. More widely known works include compilations from ancient Greece and Rome by Aristotle, Theophrastus, Dioscorides and Pliny the Elder.

The earliest compilations treated manageably small sets of species, in part because they were largely focused on medicinal plants and in part because their scope was local. By the 1600s, far-reaching European exploration and interest in global biodiversity meant that scholars were beginning to cope with much longer species lists: in 1623 Bauhin [5] recorded 6000 plant species, while in 1686 Ray [6] dealt with over 18 000. With so many species to keep track of, precision in naming became important, and a formal system began to evolve. Bauhin, Ray, and their contemporaries coined names in Latin—but (mostly) these were long, descriptive phrases rather than relatively compact binomials. For instance, in 1738 Artedi [7] named the English whiting *Gadus, dorso tripterygio, ore cirrato, longitudine ad latitudinem tripla, pinna ani prima ossiculorum trigiata* (mercifully, it is now *Merlangius merlangus*). Such unwieldy names represented attempts to accomplish two different things at once: to *name* species, providing a way to refer to them; and to *describe* (or ‘diagnose’) species, distinguishing them from their similar relatives. As the rolls of scientifically named species burgeoned, though, it became more and more difficult for names to be effective at the latter function. There were just too many similar species needing to be distinguished, and so names had to get longer and longer—and might have to be revised periodically as new biodiversity became known.

It was Linnaeus's work, in the middle third of the eighteenth century, that introduced the naming system we recognize today. The ‘innovation’ most closely associated with Linnaeus is the Latin binomial name: single-word names (each) for genus and species, as in *Homo sapiens*. Linnaeus did not, however, invent the binomial name or intend its exclusive use. Binomials had been in occasional prior use, including in 1691 by Jung [8]; and Linnaeus believed they should supplement, not replace the descriptive phrase-name [9,10]. Instead, what was truly novel about Linnaeus's nomenclatural system—what changed naming fundamentally and forever—was the decoupling of naming from description. A Linnaean binomial *can* be descriptive (consider *Anaspis flava, Carex flava, Laphria flava, Cicada flava, Motacilla flava* and *Sarracenia flava,* all of which are yellow (Latin ‘flava’) in some way), but it need not be. Linnaeus named species and genera for their geographical occurrence (*Solidago canadensis*), for their habitat (*Rubus saxatilis*), for mythological people, gods or creatures (*Papilio helena, Asclepias, Hydra*; see [9]), for people (particularly, for other botanists; for instance, *Lobelia* for Matthias de l'Obel), and more. All this was possible only because names were freed of the necessity of description. While Linnaeus did not invent the non-descriptive, binomial genus-and-species name out of thin air, he did systematize, formalize and promote it. In doing so, he made a fundamental change in the prevailing system of nomenclature that made naming the creative act it is today.

Linnaeus's naming system was quickly and almost universally adopted by Western science [11], but for a long time it was not formally codified. Over the ninteenth and twentieth centuries, detailed Codes were developed to give detailed, explicit and quasi-legal guidance to those coining and using scientific names [12]. The first was the 1843 ‘Strickland Code’ [13] covering animals; but we now have an International Code for Zoological Nomenclature (1st edition 1905; 4th edition 1999 [14]) and an International Code for Botanical Nomenclature (1st edition 1906; current edition 2018 [15]). (Three other, separate, Codes treat naming of cultivated plants, bacteria and viruses.)

The modern Codes, despite their length and complexity, place remarkably few constraints on the composition of genus and species names. The botanical Code [15] and the zoological Code [14] differ in minor technical ways; for example, tautonyms (repetition of the genus name as the species name, as in the amusing fish name *Boops boops*) are permitted for animals but not for plants. Despite this kind of variation in detail, the two codes are largely congruent in principle; unless otherwise indicated, we refer here to the zoological version. Genus and species names must each have at least two letters (Articles 11.8, 11.9; and so the winged theropod dinosaur *Yi qi* [16] will always be at least tied for the shortest scientific name). They may be formed from any language, or even from arbitrary combinations of letters that do not arise from any human language (Article 11.3), although they must be spelled using only the 26 letters of the Roman alphabet (Article 11.2; although hyphens are permitted under very limited conditions; Article 32.5.2.4.3). (In the botanical Code, both hyphens and diareses are permitted, although again under limited conditions; Articles 60.7, 60.11.) Once formed, they are often ‘Latinized’ in spelling, and are treated grammatically in some ways as if they *were* Latin—for example with suffixes to handle issues such as agreement in gender between genus and species names. That is more or less the extent of the limits on name formation: while the Codes are quite lengthy, the remainder of each covers issues such as how names are published and, when two or more names might apply to one species, which is to be used. The nomenclatural rules, then, have a lot to say about spelling and declensions and how names are to be published and applied; but they do not constrain a coiner's more fundamental choices at all. (In addition to the rules, both Codes include ‘recommendations’, but these are non-binding.) Nearly any word in any language, or none at all, can be the etymological basis of a new genus or species name.

Taxonomists have used their creative freedom under the Codes with abandon. Scientific names are still used to describe, of course, and to reference species' geography, habitat, or other biological characters. But in addition, names have been used in many other ways. Names have acknowledged contributors to science both past (the barnacle *Calantica darwini* [17]) and present (the owl *Otus bikegila*, for the Príncipe Island naturalist who spurred its discovery [18]). They have honoured scientists’ family members or partners (the spider *Tmarus manojkaushalyai* [19]). They have made rueful reference to events during the pursuit of science (the tapeworm *Phoreiobothrium perilocrocodilus* [20]). They have been used to make puns and other jokes (the tiny frog *Mini mum* [21]; the wasp *Pieza rhea* [22]). They have even, occasionally, been used to insult a namer's enemy or rival (the trilobites *Isbergia planifrons* and *I. parvula* [23,24]). This does not exhaust the list; human creativity is a deep, deep well.

## 3. Patterns in name formation: languages, etymologies, taxa and time

The fact that the etymology of scientific names is nearly free of constraint does not, of course, mean that scientific names are free of pattern. There are common tropes and practices, and uncommon ones; and these vary across taxa and through time. Patterns in name formation reveal much about the interests, personalities and cultural situations of the scientists who name species—and sometimes, of those who use the names.

### (a) Language

While the language used to form a scientific name is unconstrained by the Codes, the great majority of names that have a clear source language have always been based on either Latin or Greek. (Names that are eponymous or based on geography or host are excluded here, because they are not directly derived from any language; see next section.) ‘Non-classical’ derivations may not be common, but there are genus and species names formed from roots in a remarkable diversity of languages, including Indigenous languages of every continent (table 1). (We are concerned here with the etymological root of the name. Regardless of their linguistic origin, names are treated grammatically in some ways as if they *were* Latin, and frequently receive Latin or Greek prefixes or suffixes such as *-ensis, -oides, neo-* or *-para*.)

**Table 1.** Some examples of linguistic origins for scientific genus or species names.

scientific name	year of description	linguistic origin of underlined portion	English meaning	organism
*Alca torda*	1758	Norwegian	auk	razorbill (seabird)
*Annona cherimola*	1768	Quechua	cold seeds	cherimoya (tree)
*Beilschmiedia tawa*	1838	Te reo Māori	the tawa tree	tawa (tree)
*Bruhathkayosaurus matleyi*	1987	Sanskrit (*Bruhathkayo*) + Greek (*saurus*)	huge body + lizard	sauropod dinosaur
*Cafeteria roenbergensis*	1988	English	cafeteria, refers to indiscriminate diet	flagellate protist
*Desmognathus adatsihi*	2022	Tsalagi (Cherokee)	mother of all (refers to matrilineal inheritance)	salamander
*Guibemantis milingilingy*	2018	Malagasy	being in an uncomfortable position (refers to difficulty of collecting)	frog
*Homo naledi*	2015	Sotho	star (refers to Rising Star Cave)	early human
*Patellapis hakkiesdraadi*	2009	Afrikaans	barbed wire (describes bristles)	bee
*Prunus ilicifolia*	1841	Latin	holly-like leaf	hollyleaf cherry (tree)
*Slonik sibiricus*	1977	Russian	little elephant	weevil
*Yi qi*	2015	Mandarin	strange wing	winged theropod dinosaur

The dates in table 1 suggest a possible pattern in how languages are used to coin scientific names. An obvious hypothesis is that use of the ‘classical’ languages, Latin and Greek, reflects a kind of fusty conservatism from which taxonomists have increasingly progressed as scientific participation from the Global South has increased and as scientists everywhere have become more attuned to the recognition of human diversity. The early history is at least superficially consistent with this idea. Linnaeus himself originally wrote [25] that names should be derived from Latin or Greek, rejecting coinings from other, ‘barbarous’ languages—although he contradicted that rule elsewhere in his naming recommendations and violated it repeatedly (e.g. *Alca*, from Norwegian, table 1; *Coffea*, from Arabic; and more). (It should be noted that the meaning of ‘barbarous’ in the eighteenth century was not quite what it is today: the explicit meaning with respect to language was simply ‘other than Latin or Greek’ although more was quite likely implied [26]). Others, such as the entomologist Johan Fabricius, took stronger classical-only positions [26]. The term ‘barbarous’ remained in both botanical and zoological Codes until the mid-twentieth century (1956 and 1961, respectively), although in neither case were non-classical names ever prohibited and they have certainly been in continuous use since Linnaeus (table 1).

Have non-classical-language namings become more frequent through time? We are aware of only one study to have tested the hypothesis. Veale *et al*. [27] showed that for the flora and fauna of Aotearoa New Zealand, namings from te reo Māori and ta re Moriori have increased through time, although the 1288 so-named species they documented still represent only about 4% of Aotearoa species [26].

We tested the ‘reduced classical dominance’ hypothesis more broadly, considering the linguistic origin of 1280 species chosen from each of two large, systematic compilations of scientific names: those of Mammola *et al*. [28] for spiders and of Mlynarek *et al*. [29] for phytophagous arthropods (insects and mites). Mammola *et al*.'s compilation is close to exhaustive (for *known* spiders), being taken from the World Spider Catalogue, while Mlynarek *et al*.'s compilation represents a small (2739 species in 30 genera) sampling from global biodiversity. We first stratified each dataset by year of naming, using bins that narrowed towards the present because rates of taxonomic description have increased: 1750–1799, 1800–1849, 1850–1899, 1900–1924, 1925–1949, 1950–1974, 1975–1999 and 2000-present. We then chose names for examination randomly from those available within each temporal bin (because early bins had fewer names, samples sizes were not necessarily equal across bins) and sorted the full dataset randomly with respect to age of description. For each chosen name, we scored the linguistic origin of the species name as ‘classical’ (Latin or Greek), ‘non-classical’ (any other language, but ignoring the Latinization of non-classical forms by means of suffixes, etc.), or ‘other’ (primarily, names based on the proper name of a person, place or host species; for example, *Eurosta solidaginis* is scored ‘other’ because its name is simply that of its host, *Solidago*). Our inference about language origin relied on a combination of the describing authors' etymological explanation (when available) and our inspection of the name itself. All names were scored by an assistant who speaks and reads, to at least a basic level, English, Spanish, French, Italian, Portuguese, Korean, Japanese, Swedish, Norwegian and Danish. We are likely to have missed some non-classical origins, but have no reason to expect a year-of-description influence on these oversights. We used logistic regression to test the hypothesis that the frequency of non-classical namings increased with year of description.

**Figure 1.**
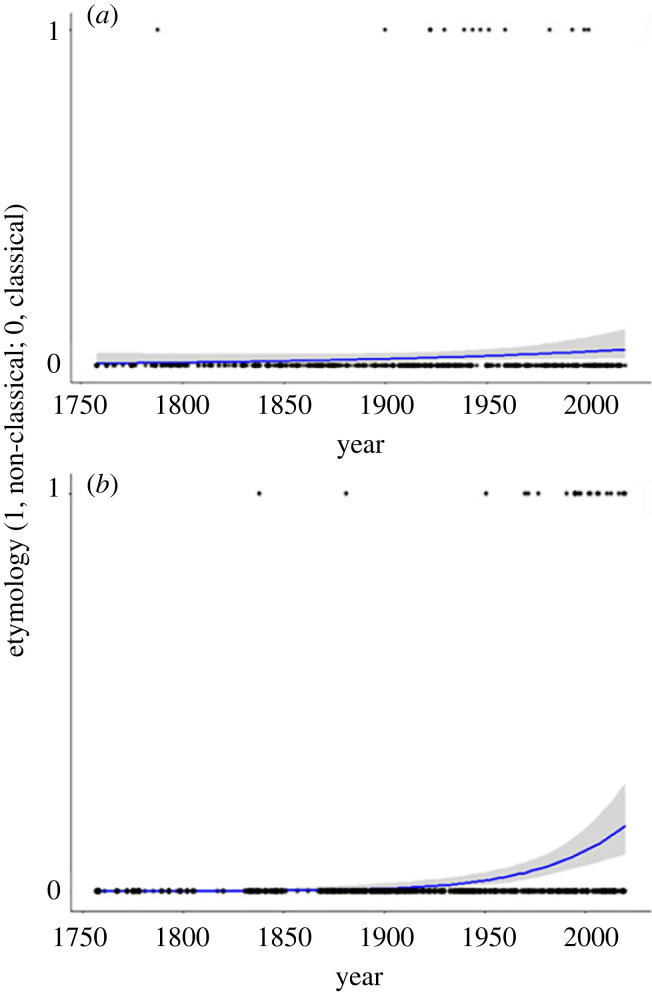
Incidence of non-classical etymologies (languages other than Latin and Greek) through time, for two large compilations of scientific names. Points are individual names; line is best logistic regression fit (with 95% confidence interval). (*a*) Plant-feeding arthropods, Z = 3.09 *p* = 0.08; names from [29]. (*b*) Spiders, Z = 37.05, *p* < 10^−8^; data from [28].

As in Veale's compilation, non-classical namings were uncommon overall: only about 1.1% (14/1280) for plant-feeding arthropods and 1.6% (21/1280) for spiders. By contrast, 43% and 56%, respectively, of namings are based on Latin or Greek (the rest either could not be classified or were scored ‘other’; for plant-feeding arthropods the latter are mostly eponymous, geographical or refer to host plants; for spiders, they are mostly eponymous or geographical). The frequency of non-classical naming increased through time for both groups (figure 1), but the increase was significant only for spiders (plant-feeding arthropods, *Z* = 3.09 *p* = 0.08; spiders, *Z* = 37.05, *p* < 10^−8^).

There is thus some evidence, for several groups of taxa, that non-classical namings have increased through time. However, there is little evidence that they have yet become routine, despite occasional claims that declining knowledge of classical languages among scientists is changing taxonomic practice. We suspect the strength of such patterns may vary among taxa, however: for instance, Chinese-derived names for dinosaurs (e.g. *Yi qi*, table 1) might show more rapid growth given the recent strength of Chinese palaeontology.

One aspect of the linguistic formation of scientific names deserves special comment. According to the nomenclatural Codes, valid names must be formed using only the 26 letters of the Roman alphabet (plus for plants, algae and fungi diareses and hyphens in limited circumstances). This constrains a namer's ability to represent the range of sounds used in human language. For example, the glottal stop that is common in languages such as Arabic, Denesilune, Hawai'ian and Samoan but rare in most dialects of English is variously recorded as ⟨ʔ⟩, ⟨7⟩, ⟨?⟩, ⟨'⟩, ⟨ʾ⟩, ⟨‘⟩, ⟨”⟩ or ⟨ء⟩ (among other symbols); but none of these characters is permissible in a scientific name. There are practical issues that weigh on the side of limiting the set of letters (and thus sounds) that can be part of a name; but these will trade off with a desire to diversify the linguistic basis of naming.

### (b) Etymology

As for source language, the nomenclatural Codes place essentially no constraint on the etymological basis for a newly coined genus of species name. However, there are broad patterns in the choices taxonomists make in constructing new names. For species names, four kinds of etymology dominate, making up over 90% of all names in published compilations for four taxa (plants in the genus *Aloe*, parasitic worms, spiders and phytophagous arthropods; table 2). Names based on morphology (or ‘descriptive’ names) are the most common in three of the four taxa, but do not constitute a majority in any of them. Three other etymologies are very common in at least one taxon: those based on a species' geographical occurrence (for example, the palm *Phoenix canariensis*, native to the Canary Islands), those based on a species’ habitat or host (such as the sand-loving plant *Aloe arenicola* or the *Solidago*-galling fly *Eurosta solidaginis*), and those based on the names of people (eponymous names, such as the huntsman spider *Heteropoda davidbowie*). All other etymologies are uncommon. There is evidence from two studies ([30] for *Aloe*, [28] for spiders) that the incidences of the four major etymologies have been changing since the late ninteenth century, in each case with names based on morphology declining sharply, and those based on geography and eponymy increasing. These changes could reflect changes in the training of those who name species, as more species are named by scientists who are not classically trained taxonomists or who have less familiarity with Latin and Greek; however, these hypotheses would be difficult to test.

**Table 2.** Etymological bases for new species names in four large compilations, from [12]. Data for spiders are estimated (and thus approximate) from fig. 1 of [28]; data for worms are estimated using combinations of subcategories from [31]. Note that totals are heavily influenced by the spider dataset, which makes up nearly 88% of the total.

basis for etymology	*Aloe*	parasitic worms (described 2000–2020)	spiders	phytophagous arthropods	totals
	per cent of species (number)	per cent of species (number)	per cent of species (number)	per cent of species (number)	per cent of species (number)
morphology	38.6 (353)	20.8 (601)	40.5 (19190)	32.7 (895)	39.1 (21039)
geography	20.0 (179)	19.0 (550)	27.2 (12868)	11.0 (300)	25.7 (3821)
habitat/host	4.0 (37)	21.3 (616)	5.8 (2750)	26.7 (731)	7.8 (4210)
eponomy	30.4 (278)	34.7 (1004)	19.4 (9157)	15.4 (422)	20.2 (10861)
other	7.4 (68)	4.2 (120)	7.1 (3360)	14.3 (391)	7.3 (3939)
total	100 (915)	100 (2891)	100 (47325)	100 (2739)	100 (53870)
citation	[30]	[31]	[28]	[29]	

A notable feature of the etymological patterns in table 2 is that they vary among the surveyed taxa (and this variation is highly significant: *p* < 10^−7^). Poulin *et al*. [31] identified similarly significant variation among clades with their parasitic-worm data. Names based on geography, for example, are relatively unusual for phytophagous arthropods (11%), while names based on habitats are uncommon in *Aloe* and for spiders (4 and 5.8%). Part of this variation is surely biological in origin: names referring to habitat/host are far more common in parasitic worms and phytophagous arthropods than they are for spiders or *Aloe*. This is unsurprising, because species in the former taxa have frequently evolved tight host-specialization, attacking a single species of host (or a few) and making a host-based name an obvious possibility. But biology seems unlikely to explain other variation in the data. For example, eponymous names are more than twice as common, but morphology-based names less common, among parasitic worms as they are among phytophagous arthropods. While parasitic worms may appear (at least to non-experts) to offer fewer distinctive morphologically features, phytophagous arthropod lineages are replete with morphologically cryptic species—making it difficult in both groups to base names on species-diagnostic morphological traits. Much etymological variation may arise instead from cultural variation in naming practice among taxonomists or groups of taxonomists. Veale *et al*. [27] have pointed to the contributions of two individual taxonomists (the arachnologist Ray Forster and the malacologist Arthur Powell) who strongly influenced the linguistic source of species names in the New Zealand fauna (both were prolific namers of species who frequently used names deriving from te reo Māori). Poulin *et al*. [31] similarly identified individual taxonomists of outsized influence on the etymological basis of names in particular groups of parasitic worms (that is, at a finer taxonomic level than seen in table 2). But this variation in practice is not likely to lie entirely at the level of individual scientists; similar variation is likely to occur among geographic regions among the taxonomic community, among academic lineages (if students are influenced in naming practice by their advisors) and more [28,31].

### (c) Eponomy

Eponymous names (those formed from the name of a person, whether real or fictional) represent a particularly interesting etymological class. Linnaeus's freeing of naming from description made them a possibility, and he outlined at some length (in his 1737 *Critica Botanica* [32] and elsewhere) how and when eponymous naming should be done. Linnaeus was particularly interested in coining generic names from the names of other botanists, and argued that this should be done only for the best of reasons, to honour worthy colleagues, and only by the ‘greatest botanists’ of ‘mature age’ [32]. He then promptly, repeatedly and enthusiastically violated these principles. For example, he named *Linnaea* after himself [24], and other genera, unkindly, after botanists whom he considered inferior: the weedy *Siegesbeckia* after Johann Siegesbeck, and *Commelina* after Jan, Caspar, and Casparus Commelin (for Linnaeus, *Commelina*'s two showy petals were for Jan and Caspar, and the third inconspicuous one for Casparus, who ‘died before accomplishing anything in botany’; the name was originally suggested by Charles Plumier but adopted and explained by Linnaeus [33]). Eponymous namings have proliferated since and are increasing in frequency (table 2; [28,30]), and often receive considerable popular attention. Heard [24] has recently explored the history and practice of eponymous naming in detail.

Who are the people whose names are used in eponymy? For the first 150 years or so, most eponymous names referred to scientists or collectors [28]—although there were certainly exceptions, as in the mynah *Mino anais*, named poignantly (as *Sericulus anais*) in 1839 by René Lesson [34] for his daughter, deceased at the age of 11. More recently, the roster of eponyms has diversified greatly. Species have been named for partners and family members of the namers (e.g. the crab spider *Tmarus manojkaushalyai*, named for the husband of its discoverer [19]); for politicians, actors, musicians and other celebrities (e.g. the horsefly *Scaptia beyoncae*; [35]); for someone who has paid for the privilege (e.g. the cockroach *Xeroblatta berenbaumae*; [36]); or even (but very rarely) for the namers themselves (‘ego naming’; [24]). Few studies have systematically assessed the incidence of these different eponymic choices, but Poulin *et al*. [31] provided a breakdown for parasitic worms (table 3). At least 80% of eponyms are scientific contributors of some sort (first, second and fourth rows of table 3), and among the rest the great majority are partners or family members of the namers. We assume that efforts to make similar compilations for other taxa would turn up the same kind of cross-taxon variation we demonstrate in table 2.

**Table 3.** Source of eponymic names in parasitic worms (data from Poulin *et al*. [31]). Several entries are upper limits because some of Poulin's categories are not precisely eponymic: for instance, fictional human characters are combined in a single category with mythical creatures. The total of 1004 is our best estimate.

type of eponym	number of species
eminent scientist	596
provider of technical/logistic support	143
partner or family member	<140
collector/provider of type material	58
local cultural icon	<28
philanthropist or administrator	24
mentor or personal supporter	22
celebrity	13
fictional character	<8
total	∼1004

The creative nature of naming means that the set of names that are coined reflects the cultural backgrounds and attitudes, and the personalities, of the namers. We are perhaps accustomed to praising creativity as always a virtue, but of course it need not be. Just as one example, poetry can be used to give voice to antisemitism (e.g. [37,38], but see [39] and compare [40]). Similarly, creativity in naming can be used poorly, and the practice of eponymous naming in particular has come under intense criticism, mostly for two related reasons. First, the *set* of people ‘honoured’ (although honour is not always intended) by eponymous names does not yet do a good job of representing the diversity of the human species. Second, some of the *individual* people chosen for eponymy are at least in hindsight regrettable choices. We summarize each of these issues but note that a full discussion is beyond the scope of this paper; we refer readers to the burgeoning literature.

Compilations of species named eponymously to date demonstrate a strong, unfortunate, but unsurprising pattern: most eponyms are white males from the Global North. For example, in *Aloe* Figueiredo & Smith [30] estimated that only 13% of eponyms were female (we note here the usual, and important, caveats about the impossibility of inferring gender with complete accuracy from names alone and about the non-binary nature of human gender). Of that 13%, nearly half were identified as partners or relatives of the namers rather than as people being honoured for contributions to science. Similar estimates are available for the vascular flora of New Caledonia (6% female eponyms [41]), freshwater fishes of North Carolina (14% [42]), birds (19% for species named between 1950 and 2019 [43]) and parasitic worms (19% among ‘eminent scientist’ eponyms for species named between 2000 and 2020 [31]). Poulin *et al*. [31] found no recent temporal trend in the representation of women among eponyms; Pillon [41], in contrast, demonstrated a strong trend to increasing representation of women since the mid-twentieth century. A similar pattern exists in representation of local and Indigenous eponyms [24] versus those from the Global North. For the New Caledonian flora, only 7% of eponyms are New Caledonia-born, although the fraction is much higher in species described since the mid-twentieth century ([41] this compilation did not attempt to distinguish Indigenous versus non-Indigenous New Caledonians). For birds, species named since 1950 have nearly all (96%) been from the Global South, but 68% of eponyms are from the Global North [43]. The fraction of bird species named for *local* eponyms is, however, increasing through time [43]. Among North Carolina freshwater fishes, 28 species are named for individual people, none of them apparently Indigenous [42] (a single species (the Sandhills Chub *Semotilus lumbee* is named for an Indigenous people as a whole). We expect that compilations for other taxa will show similar patterns (e.g. [44]), although the emphasis on birds, fish, and other taxa for which most species have long since been described and named blunts the possibility for recent change in eponymous representation.

Considerable attention has recently been drawn to species named for people whose actions or character have been unsavoury or worse (e.g. [24,42,44–46]). The eponyms in question run the gamut from those accused of minor peccadillos to some of the most evil human beings to have lived (the canonical example being the eponym behind the Slovenian cave beetle *Anophthalmus hitleri*). There are no provisions in the nomenclatural Codes prohibiting such namings, and no provisions specifically allowing for names to be suppressed or changed based on the distastefulness of their eponyms. Currently, the zoological Code recommends, but does not require, the avoidance of names that are inappropriate or that might cause offense (Recommendation 25C). The botanical Code goes further, dictating that ‘a legitimate name must not be rejected merely because it, or its epithet, is inappropriate or disagreeable’ (Article 51.1). But the Codes could be amended, and there have been both informal and formal proposals to do so (e.g. [42,45,47,48]). Should this happen, and if it does, how should we proceed to deal with unsavoury eponymous names? There are likely hundreds of thousands of eponymous names, and part of the problem is that current processes for changing names are complex, bureaucratic, and time-consuming. We suspect that it is necessary that they be so. A more important part of the problem is that the eponyms behind these species names (like the human species from which they are sampled) embody all manner and combinations of virtue and vice. Some have argued that it will be relatively easy to reach agreement on which names should be changed and which should not (e.g. [45]). Others have argued that in part because such agreement cannot be reached, no names should be changed (beyond a very few exceptions that could be managed under the current Codes; e.g. [49,50]). Still others have argued that *all* eponymous names should be changed and no further ones should be bestowed (e.g. [44]). Discussion continues, with nothing remotely resembling a consensus on the way forward—as is evident from a set of six opinion pieces, recently published together, that run the gamut from arguments that eponymous names have no place whatsoever in science to arguments that proposals to ban eponyms amount to a new form of colonialist imposition on scientists from the Global South ([44,51–55], and see also [56]).

## 4. When creativity shapes science

While we contend that on its own, the creativity of scientists on view through scientific naming is fascinating and deserving of continued study, there is more to the story. In multiple ways, creativity in naming has consequences for the science we do, influencing its resourcing, its public communication, and even its direction. We explore each of these influences, although for each, rigorous study is in its infancy.

### (a) Scientific naming and funding for research and conservation

Perhaps the most direct and obvious link between naming and the pursuit of science lies in the potential for naming to generate funding for research. The discovery and description of new (to science) species is critical to our understanding of Earth's biodiversity, and has considerable urgency given the ongoing extinction crisis: many species are doubtlessly being lost even before they are known to science [57–59]. However, funding opportunities for species-discovery science are shamefully limited, especially compared with higher-visibility sciences such as astronomy, particle physics, or oncology. In this context, it is not surprising that some taxonomists have found novel funding potential in scientific naming.

The best-established example of funding through naming is BIOPAT, or the Patenschaften für biologische vielfalt (‘Sponsorships for Biodiversity’; https://biopat.de). Several research institutions collaborate to run BIOPAT, including the Senckenberg Centre for Biodiversity Research, the Alexander Koenig Research Museum, and the Bavarian State Collection of Zoology. The program matches interested donors with researchers who are naming new species; in return for a donation of at least €2600, the donor chooses a species name and the researcher adopts it in publishing the new species' description. Since its inception, the program has arranged the naming of 190 species and received about €650 000 in corresponding donations (Jörn Köhler, 27 June 2023 and Claus Baetke, 1 July 2023, personal communications). Half of each donation is routed to the describing taxonomist's institution, designated for research support, and the other half is routed to a granting program supporting biodiversity research and conservation in the region where the species occurs. The great majority of donors have opted for eponymous naming, requesting species named for themselves (e.g. *Boophis fayi*, for Andreas Norbert Fay [60]), their family members (*Euspondylus caideni*, for a son of donor Stan Vlasimsky [61]), or other figures they love or admire (*Polystachya anastacialynae*, for the pop singer Anastacia [62]). However, there are also species named for corporate donors (*Sinopoda scurion* [63]) and a few non-eponymous namings (*Moraea vuvuzela*; [64]). Other organizations have mounted similar fundraising programs, including the Scripps Institute of Oceanography (https://scripps.ucsd.edu/giving/name-new-species) and the Australian Museum [65], and individual scientists have occasionally offered namings by donation for particular species (e.g. [36]).

There is nothing new about naming recognizing donors, except perhaps its transparency: for centuries taxonomists have named species after their patrons, or sponsors of expeditions. (Many, especially during the Victorian boom in natural-history exploration and collecting, have also sold collected specimens to fund their work [66,67]). However, some have objected to naming sales, on at least two different grounds (e.g. [68]). First, it may feel unseemly to ‘commercialize’ naming, especially if donations are used for personal financial gain rather than for research. Second, it might incentivize overzealous description of ‘new’ species and thus degrade the taxonomic literature with a flood of spurious descriptions and unnecessary junior synonyms. Fortunately, after more than two decades of organized fundraising through naming, there is no evidence that either of these fears has been realized. We join Evenhuis (quoted in [65]) in wishing that the funding landscape for taxonomy were such that the debate were unnecessary.

Namings-for-donation schemes have had similar, but much larger, impacts in conservation. Two significant fundraising events demonstrate the potential. In 2007, an auction of naming rights for 10 western Pacific fishes raised over USD $2 000 000 for ocean conservation in Indonesia [69], and in 2006, Wallace *et al*. [70] named a Bolivian monkey *Callicebus aureipalatii* after the online casino GoldenPalace.com won a Wildlife Conservation Society auction with a USD $650 000 donation to FUNDESNAP (the Bolivian Foundation for the Development of the National Protected Area System [71]). Of course, conservation and biodiversity research are often tightly linked, and conservation programs and infrastructure supported by these donations have also facilitated continued local research.

### (b) Scientific naming and public attention to science

New telescopes, the latest climate-change data, and experimental cancer treatments often make the (now metaphorical) front pages in popular media. The science of species discovery is, unfortunately, much more obscure. Creativity in naming may offer a tool to rectify this, with some new scientific names seemingly designed to garner public attention. What kind of names might those be, and how effective is the strategy?

One possibility is the use of naming to draw public attention to a species, and to a conservation threat that might imperil it. Bernal *et al*. [72] took this approach with the naming of the palm *Aiphanes argos* after the Colombian company Grupo Argos. That company had planned to build a hydroelectric dam on the Rio Samaná Norte—a dam that would have flooded over 90% of the palm's habitat. The naming was an explicit attempt to raise awareness of the need to protect the habitat [73]. There was indeed a media uproar, including discussion of the palm's naming, and the dam was never built. However, it is unclear how important the naming was in derailing Grupo Argos' plan, and it is difficult to conceive of an analysis that could shed much light.

Naming might also draw public attention to the science of species discovery itself. In particular, the naming of species for celebrities [24] seems like a strategy tailor-made for generating public attention to species discovery. Eponymous names have been coined for musicians (the fern *Gaga monstraparva* [74]), actors (the beetle *Agra katewinsletae* [75]), filmmakers (the alga *Euthora timburtonii* [76]), athletes (the wasp *Diolchogaster ichiroi* [77]), politicians (the lichen *Ocellularia jacinda-arderniae* [78]), activists (the frog *Pristimantis gretathunbergae* [79]), novelists (the fossil sea turtle *Psephophorus terrypratchetti* [80]) and more. Blake *et al*. [81] have shown that species with such names attract more Wikipedia page views than their close relatives with non-celebrity names—with the effect being especially strong in less charismatic taxa such as invertebrates and amphibians, and weaker to undetectable for birds and mammals. Of course, some celebrities are viewed negatively, and the additional attention garnered by species associated with them might not be a good thing (as an analogy, there is some evidence that human sentiment associated with species common names can affect opinions about conservation worth [82]; but see [83,84]).

Many celebrity-named species receive coverage in popular media, which might contribute to the public attention they, and thus species discovery more generally, receive. But does celebrity naming actually drive media attention?

No study has yet attempted test this hypothesis, or even to quantify the incidence of celebrity namings in popular media. We take the first steps in such an analysis here. Using the PressReader app (PressReader Inc., Richmond, Canada), we searched for articles from newspapers and magazines from 2017 to 2022 with the terms ‘new’ and ‘species’. This resulted in 108 000 articles. We checked articles from this compilation to determine whether they included the scientific name for a newly described species, working the order in which articles were listed in the search results and continuing until we had 300 new-species mentions. We scored the etymology of these new species as either being eponymous and based on a celebrity, or not (any other naming, whether or not eponymous). A ‘celebrity’ was a person whose name is widely recognizable for reasons other than scientific achievement; we expected some difficulty classifying people as ‘celebrities’ or not, but in fact had none. For each new species, we then searched PressReader to determine how many *other* magazines and newspapers ran stories about that new species. We take this measure of ‘journalistic spread’ as an indicator of media attention paid to the new species.

Somewhat to our surprise, celebrity namings constitute a very small fraction of media reports on new species descriptions: about 4.3% (13/300; table 4). However, species with celebrity names appear in significantly more media stories than species with other naming etymologies (over twice as many: x̅_celebrity_ = 7.0; x̅_other_ = 2.7; *t* = 2.47, *p* = 0.01 by permutation *t*-test). If anything, our analysis should underestimate the influence of celebrity naming on attention, because the existence of a species in our dataset—regardless of its name—is conditioned on its appearance in at least one indexed article. A more powerful analysis could take either of two approaches. One could compare, for some diverse taxon, the proportion of celebrity names among media-covered new species and all new species. Alternatively, one could find (independently of media coverage) pairs of recently described congeners for which one is named for a celebrity and the other is not, and then use a contingency-table test to ask whether celebrity names are more likely to receive media coverage. Such an analysis would be fascinating, but must await future work.

**Table 4.** Journalistic ‘spread’ of stories referring to new species named for celebrities, versus other new species. In all cases, we located a single news story about a newly named species, and tabulated the number of *additional* stories published about the same species as an indicator of media interest. Entries are alphabetical by celebrity surname (with apologies to Lady Gaga's Little Monsters, who will doubtless protest that ‘Gaga’ is not really a surname).

species name	year of description	celebrity	organism	number of additional stories
*Hemiandrus jacinda*	2021	Jacinda Ardern	weta (insect)	1
*Pristimantis attenboroughi*	2017	David Attenborough	frog	0
*Uvariopsis dicaprio*	2022	Leonardo Dicaprio	tree	9
*Duvalius djokovici*	2022	Novak Djokovic	beetle	20
*Travunijana djokovici*	2021	Novak Djokovic	snail	3
*Kaikaia gaga*	2020	Lady Gaga	treehopper (insect)	17
*Andrena hadfieldi*	2020	Chris Hadfield	bee	16
*Jotus karllagerfeldi*	2019	Karl Lagerfeld	spider	4
*Daptolestes leei*	2020	Stan Lee	fly	4
*Sointharus barackobamai*	2018	Barack Obama	spider	1
*Opacuincola gretathunbergae*	2021	Greta Thunberg	snail	0
*Craspedotropis gretathunbergae*	2020	Greta Thunberg	snail	16
*Thunberga greta*	2020	Greta Thunberg	spider	0
all non-celebrity namings (*n* = 287)		—	various	2.7

### (c) Scientific naming and the direction of research studies

Could creativity in scientific naming have consequences not just for the funding and communication of science, but even for whether and how scientists study the named species? This may seem a far-fetched hypothesis, but there is strong evidence that it is true in at least one case. Mlynarek *et al*. [29] considered the case of phytophagous arthropods (insects and mites), which are often named in reference to their plant hosts (table 2, fourth column). This is likely at least in part because many, but not all, species have evolved considerable host specialization, feeding on one or a few plant species. This eco-evolutionary phenomenon has also driven a large body of work on the process of host-associated differentiation (HAD), in which an initially generalist or oligophagous species evolves tighter host specialization, resulting in a complex of species or host races associated with individual hosts. Many phytophagous arthropods have been screened, usually by population genetics, for evidence of HAD—but there are far too many species for us to screen them all. Which species, of all the possibilities, are studied this way? Mlynarek *et al*. [29] showed that arthropods named after their host are more than twice as likely to be studied as possible cases of HAD. (It was not the case that the species were named because they were known to be specialists—the naming always preceded the HAD tests, often by a century or more). This result may not be entirely surprising, as a researcher learning a species' host-based name might find their attention subconsciously steered to the question of their diet specialization. The *strength* of the effect, however, is remarkable.

Mlynarek *et al*.'s [29] study is the first, to our knowledge, to have investigated the influence of species' names on scientific attention. Given that electronic databases of species names and of our literature are improving, along with the information technology to query them, the time is ripe for further such work. Many questions suggest themselves. Might species with names that are long and difficult to spell or pronounce find themselves understudied? One of us (SBH) has published repeatedly on the gallmaking moth *Gnorimoschema gallaesolidaginis* (e.g. [85–88]) and hopes one day to type it correctly on the first try; the newly described myxobacterium *Myxococcus llanfairpwllgwyngyllgogerychwyrndrobwllllantysiliogogogochensis* [89] offers a next-level challenge. Might species named eponymously for odious people or containing racial epithets [47,48,90] attract less scientific attention because researchers are reluctant to use the names, or more because such species are widely discussed and memorable? If work on such species is done, might it be cited less, or cited more, for the same reasons? There are many reasons, beyond the importance of the data, why some species receive more scientific attention than others [91,92]. Adding the etymology of scientific names to this list is an acknowledgement that cultural factors and the psychology of scientists are important factors in the pursuit of our scientific enterprise.

## 5. Conclusion

Many scientists, we suspect, pay little attention to the scientific names of the organisms our fields seek to understand—viewing them at best as necessary but uninteresting labels, at worst as annoyances. We have argued that, because the nomenclatural Codes impose surprisingly few constraints on the formation of a new scientific name, naming is one of the most creative acts in all the sciences. The results of unleashing scientists’ creativity have been fascinating, with species bearing scientific names coming from many source languages and with a remarkable variety of etymologies. Scientific names (like the scientists who confer them) can be beautiful, irritating, amusing, poignant, provocative or infuriating. They raise important issues of social justice and equity in the history and practice of science. Finally, they can apparently have a role in directing our scientific attention to study at least some questions in *this* species rather than *that* one. All this is an excellent reminder that science and scientists are socially situated, and that human nature will always be inextricable from our pursuit of knowledge.

## Data Availability

All data and R scripts are provided as electronic supplementary material [93].

## References

[1] Heard SB. 2014 On whimsy, jokes, and beauty: can scientific writing be enjoyed? Ideas Ecol. Evol. **7**, 64-72.

[2] Heard SB. 2022 The scientist's guide to writing : How to write more easily and effectively throughout your scientific career, 2nd edn. Princeton, NJ: Princeton University Press.

[3] Böck B. 2015 Shaping texts and text genres: on the drug lore of Babylonian practitioners of medicine. Aula Orient. **33**, 21-37.

[4] Nugent-Head J. 2014 The first Materia Medica: The Shen Nong Ben Cao Jing. J. Chin. Med. **104**, 24-28.

[5] Bauhin C. 1623 Pinax theatri botanici (illustrated exposition of plants). Basel, Switzerland: Sumptibus & typis Ludovici Regis.

[6] Ray J. 1686 Historia plantarum. London, UK: Typis Mariæ Clark, prostant apud Henricum Faithorne.

[7] Artedi P. 1738 Ichthyologia sive opera omnia de piscibus, scilicet: bibliotheca ichthyologica. Philosophia ichthyologica. Genera piscium. Synonymia specierum. Descriptiones specierum. Omnia in hoc genere perfectiora, quam antea ulla. Leiden, The Netherlands: Lugduni Batavorum, apud Conradum Wishoff.

[8] Jung J. 1691 Historia vermium. Hamburg, Germany: Typis Brendekianis.

[9] Heller JL. 1945 Classical Mythology in the *Systema Naturae* of Linnaeus. Trans. Proc. Am. Philol. Assoc. **76**, 333-357. (10.2307/283345) 11618222

[10] Stearn WT. 1959 The background of Linnaeus's contributions to the nomenclature and methods of systematic biology. Syst. Zool. **8**, 4-22. (10.2307/2411603)

[11] Winston JE. 2018 Twenty-first century biological nomenclature: The enduring power of names. Integr. Comp. Biol. **58**, 1122-1131. (10.1093/icb/icy060) 30113637

[12] Heard SB. 2023. The name of the rose (and everything else): how codes and practices in naming biological species reflect cultural identities. In Names, naming and the law: onomastics, identity, power, and policy (ed. IM Nick), 1st edn. New York, NY: Routledge.

[13] Strickland HE. 1843 Report of a committee appointed to ‘consider of the rules by which the nomenclature of zoology may be established on a uniform and permanent basis’. In Report of the 12th meeting of the British Association for the Advancement of Science [1842], pp. 105-121. London, UK: John Murray.

[14] International Commission on Zoological Nomenclature. 1999 International code of zoological nomenclature, 4th edn. London, UK: Natural History Museum (London) Publications.

[15] Turland NJ et al. 2018 International code of nomenclature for algae, fungi, and plants (Shenzhen code) adopted by the nineteenth international botanical congress Shenzhen, China, July 2017. Glashütten: Koeltz Botanical Books.

[16] Xu X et al. 2015 A bizarre Jurassic maniraptoran theropod with preserved evidence of membranous wings. Nature **521**, 70-73. (10.1038/nature14423) 25924069

[17] Jones DS, Hosie AM. 2009 A new species of *Calantica* from Western Australian waters (Thoracica: Scalpellomorpha: Calanticidae). Rec. West. Aust. Mus. **25**, 239-246. (10.18195/issn.0312-3162.25(3).2009.239-246)

[18] Melo M et al. 2022 A new species of scops-owl (Aves, Strigiformes, Strigidae, *Otus*) from Príncipe Island (Gulf of Guinea, Africa) and novel insights into the systematic affinities within *Otus*. ZooKeys **2022**, 1-54. (10.3897/zookeys.1126.87635) PMC9836643 36763062

[19] Arachchi IS, Benjamin SP. 2019 Twigs that are not twigs: phylogenetic placement of crab spiders of the genus *Tmarus* of Sri Lanka with comments on the higher-level phylogeny of Thomisidae. Invertebr. Syst. **33**, 575-595.

[20] Caira JN, Richmond C, Swanson J. 2005 A revision of *Phoreiobothrium* (Tetraphyllidea: Onchobothriidae) with descriptions of five new species. J. Parasitol. **91**, 1153-1174. (10.1645/GE-3459.1) 16419764

[21] Scherz MD et al. 2019 Morphological and ecological convergence at the lower size limit for vertebrates highlighted by five new miniaturised microhylid frog species from three different Madagascan genera. PLoS ONE **14**, e0213314. (10.1371/journal.pone.0213314) 30917162 PMC6436692

[22] Evenhuis NL. 2002 *Pieza*, a new genus of microbombyliids from the New World (Diptera: Mythicomyiidae). Zootaxa **36**, 1-28. (10.11646/zootaxa.36.1.1)

[23] Warburg E. 1925 The trilobites of the Leptrena limestone in Dalarne: with a discussion of the zoological position and the classification of the trilobita. Bulletin of the Geological Institutions of the University of Uppsala **17**, 1-446.

[24] Heard SB. 2020 Charles darwin's barnacle and david bowie's spider : How scientific names celebrate adventurers, heroes, and even a Few scoundrels. New Haven, CT: Yale University Press.

[25] Linnaeus C. 1721 Philosophia botanica. Stockholm, Sweden: G. Keiswetter.

[26] Galbreath R. 2021 Why have so few Māori or Moriori names been used in taxonomic description? N. Z. J. Ecol. **45**, 3429.

[27] Veale AJ et al. 2019 Using te reo Māori and ta re Moriori in taxonomy. N. Z. J. Ecol. **43**, 1. (10.20417/nzjecol.43.30)

[28] Mammola S, Viel N, Amiar D, Mani A, Hervé C, Heard SB, Fontaneto D, Pétillon J. 2023 Taxonomic practice, creativity and fashion: what's in a spider name? Zool. J. Linn. Soc. **198**, 494-508. (10.1093/zoolinnean/zlac097)

[29] Mlynarek JJ, Cull C, Parachnowitsch AL, Vickruck JL, Heard SB. 2023 Can species naming drive scientific attention? A perspective from plant-feeding arthropods. Proc. R. Soc. B **290**, 20222187. (10.1098/rspb.2022.2187) PMC9904940 36750196

[30] Figueiredo E, Smith GF. 2010 What's in a name: epithets in *Aloe* L. (Asphodelaceae) and what to call the next new species. Bradleya **2010**, 79-102. (10.25223/brad.n28.2010.a9)

[31] Poulin R, McDougall C, Presswell B. 2022 What's in a name? Taxonomic and gender biases in the etymology of new species names. Proc. R. Soc. B **289**, 20212708. (10.1098/rspb.2021.2708) PMC9091844 35538778

[32] Linnaeus C. 1737 Critica botanica. Leiden, The Netherlands: Lugduni Batavorum: Apud Conradum Wishoff.

[33] Stearn WT. 1996 Stearn's dictionary of plant names for gardeners. London, UK: Cassall's.

[34] Lesson RP. 1839 Oiseaux rares ou nouveaux de la collection du Docteur Abeillé à Bordeaux. Rev. Zool. Par Société Cuvierienne **2**, 40-43.

[35] Lessard BD, Yeates DK. 2011 New species of the Australian horse fly subgenus *Scaptia* (*Plinthina*) Walker 1850 (Diptera: Tabanidae), including species descriptions and a revised key. Aust. J. Entomol. **50**, 241-252. (10.1111/j.1440-6055.2011.00809.x) 23270148

[36] Evangelista D. 2014 Vengeful taxonomy: your chance to name a new species of cockroach. *Entomol. Today*. See http://entomologytoday.org/2014/03/20/vengeful-taxonomy-your-chance-to-name-a-new-species-of-cockroach/ (accessed 9 August 2023).

[37] Dowthwaite J. 2018 ‘Crime ov two centuries': anti-Semitic conspiracy theory as a narrative arc in Ezra Pound's ‘Cantos’. Am. Stud. **62**, 413-436.

[38] Julius A. 2003 T. S. Eliot, anti-Semitism, and literary form. New edition with a preface and a response to the critics. London, UK: Thames and Hudson.

[39] Schuchard R. 2003 Burbank with a Baedeker, Eliot with a cigar: American intellectuals, anti-Semitism, and the idea of culture. Modernism/modernity **10**, 1-26. (10.1353/mod.2003.0021)

[40] Perloff M. 2003 A response to Ronald Schuchard. Mod./Mod. **10**, 51-56. (10.1353/mod.2003.0023)

[41] Pillon Y. 2021 The inequity of species names: the flora of New Caledonia as a case study. Biol. Conserv. **253**, 108934. (10.1016/j.biocon.2020.108934)

[42] Tracy BH. 2022 What's in a fish species name and when to change it? Fisheries **47**, 337-345. (10.1002/fsh.10750)

[43] DuBay S, Droguett DHP, Piland NC. 2022 Global inequity in scientific names and who they honor. *BioRxiv* 2020. (10.1101/2020.08.09.243238)

[44] Guedes P et al. 2023 Eponyms have no place in 21st-century biological nomenclature. Nat. Ecol. Evol **7**, 1157-1160. (10.1038/s41559-023-02022-y) 36914774

[45] Hammer TA, Thiele KR. 2021 (119–122) Proposals to amend Articles 51 and 56 and Division III, to allow the rejection of culturally offensive and inappropriate names. Taxon **70**, 1392-1394. (10.1002/tax.12620)

[46] Smith GF, Figueiredo E. 2022 ‘Rhodes-’ must fall: Some of the consequences of colonialism for botany and plant nomenclature. Taxon **71**, 1-5. (10.1002/tax.12598)

[47] Gillman LN, Wright SD. 2020 Restoring indigenous names in taxonomy. Commun. Biol. **3**, 1-3. (10.1038/s42003-020-01344-y) 33097807 PMC7584613

[48] Smith GF, Figueiredo E. 2021 (126) Proposal to add a new Article 61.6 to permanently and retroactively eliminate epithets with the root caf[e]r- or caff[e]r- from the nomenclature of algae, fungi and plants. Taxon **70**, 1395-1396. (10.1002/tax.12622)

[49] Mosyakin SL. 2022 If ‘Rhodes-’ must fall, who shall fall next? Taxon **71**, 249-255. (10.1002/tax.12659)

[50] Ceríaco LMP et al. 2023 Renaming taxa on ethical grounds threatens nomenclatural stability and scientific communication: communication from the International Commission on Zoological Nomenclature. Zool. J. Linn. Soc. **197**, 283-286. (10.1093/zoolinnean/zlac107)

[51] Antonelli A et al. 2023 People-inspired names remain valuable. Nat. Ecol. Evol. **7**, 1161-1162. (10.1038/s41559-023-02108-7) 37337005

[52] Mabele MB, Kiwango WA, Mwanyoka I. 2023 Disrupting the epistemic empire is necessary for a decolonial ecology. Nat. Ecol. Evol. **7**, 1163. (10.1038/s41559-023-02105-w) 37337004

[53] Orr MC et al. 2023 Inclusive and productive ways forward needed for species-naming conventions. Nat. Ecol. Evol. **7**, 1168-1169. (10.1038/s41559-023-02103-y) 37337001

[54] Roksandic M, Musiba C, Radović P, Lindal J, Wu X-J, Figueiredo E, Smith GF, Roksandic I, Bae CJ. 2023 Change in biological nomenclature is overdue and possible. Nat. Ecol. Evol. **7**, 1166-1167. (10.1038/s41559-023-02104-x) 37337002

[55] Thiele KR. 2023 Some, but not all, eponyms should be disallowed. Nat. Ecol. Evol. **7**, 1170. (10.1038/s41559-023-02106-9) 37337003

[56] Pethiyagoda R. 2023 Policing the scientific lexicon: the new colonialism? Megataxa **10**, 20-25. (10.11646/megataxa.10.1.4)

[57] Theng M, Jusoh WFA, Jain A, Huertas B, Tan DJX, Tan HZ, Kristensen NP, Meier R, Chisholm RA. 2020 A comprehensive assessment of diversity loss in a well-documented tropical insect fauna: almost half of Singapore's butterfly species extirpated in 160 years. Biol. Conserv. **242**, 108401. (10.1016/j.biocon.2019.108401)

[58] Moura MR, Jetz W. 2021 Shortfalls and opportunities in terrestrial vertebrate species discovery. Nat. Ecol. Evol. **5**, 631-639. (10.1038/s41559-021-01411-5) 33753900

[59] Liu J, Slik F, Zheng S, Lindenmayer DB. 2022 Undescribed species have higher extinction risk than known species. Conserv. Lett. **15**, e12876. (10.1111/conl.12876)

[60] Köhler J, Glaw F, Rosa GM, Gehring P-S, Pabijan M, Andreone F, Vences M. 2011 Two new bright-eyed treefrogs of the genus *Boophis* from Madagascar. Salamandra **47**, 207-221.

[61] Köhler G. 2003 Two new species of *Euspondylus* (Squamata: Gymnophthalmidae) from Peru. Salamandra **39**, 5-20.

[62] Fischer E, Killmann D, Lebel J-P, Delepierre G. 2010 *Polystachya anastacialynae*, eine neue Art aus dem Nyungwe Nationalpark, Ruanda - *Polystachya anastacialynae* - a new species from Nyungwe National Park, Rwanda. Orchid. **61**, 240-244.

[63] Jäger P. 2012 Revision of the genus *Sinopoda* Jäger, 1999 in Laos with discovery of the first eyeless huntsman spider species (Sparassidae: Heteropodinae). Zootaxa **3415**, 37-57. (10.11646/zootaxa.3415.1.3)

[64] Goldblatt P, Manning JC. 2010 *Moraea intermedia* and *M. vuvuzela* (Iridaceae-Iridoideae), two new species from western South Africa, and some nomenclatural changes and range extensions in the genus. Bothalia **40**, 147-153. (10.4102/abc.v40i2.204)

[65] Trivedi BP. 2005 What's in a species’ name? More than $450,000. Science **307**, 1399. (10.1126/science.307.5714.1399) 15746404

[66] Honigsbaum M. 2001 The fever trail: in search of the cure for malaria. New York, NY: Macmillan.

[67] Heard SB. 2018 Entrepreneurship in Victorian botany: did you know that was a thing? *Scientist Sees Squirrel*. See https://scientistseessquirrel.wordpress.com/2018/06/04/entrepreneurship-in-victorian-botany-did-you-know-that-was-a-thing/.

[68] Minelli A, Kraus O, Tubbs PK. 2000 Names for cash. Science **287**, 1203-1204. (10.1126/science.287.5456.1203d) 10712151

[69] Eilperin J. 2007 Auction to name fish species nets $2 million for conservation. *Washington Post*, 22 September. See https://birdsheadseascape.com/download/media/Sept07%20Washington%20Post%20Blue%20Auction%20Results.pdf

[70] Wallace RB, Gómez H, Felton A, Felton AM. 2006 On a new species of titi monkey, Genus *Callicebus* Thomas (Primates, Pitheciidae), from western Bolivia with preliminary notes on distribution and abundance. Primate Conserv. **2006**, 29-39. (10.1896/0898-6207.20.1.29)

[71] Montanari S. 2019 Taxonomy for sale to the highest bidder. *Undark Mag*. See https://undark.org/2019/04/10/nomenclature-auctions-bidder/ (accessed 1 September 2023).

[72] Bernal R, Hoyos-Gómez SE, Borchsenius F. 2017 A new, critically endangered species of *Aiphanes* (Arecaceae) from Colombia. Phytotaxa **298**, 65-70. (10.11646/phytotaxa.298.1.6)

[73] Bernal R, Borchsenius F, Hoyos-Gómez SE, Manrique HF, Sanín MJ. 2019 A revision of the *Aiphanes parvifolia* complex (Arecaceae). Phytotaxa **411**, 275-292. (10.11646/phytotaxa.411.4.3)

[74] Li F-W, Pryer KM, Windham MD. 2012 *Gaga*, a new fern genus segregated from *Cheilanthes* (Pteridaceae). Syst. Bot. **37**, 845-860. (10.1600/036364412X656626)

[75] Erwin TL. 2002 The beetle family Carabidae of Costa Rica: twenty-nine new species of *Agra* Fabricius 1801 (Coleoptera: Carabidae, Lebiini, Agrina). Zootaxa **119**, 1-68. (10.11646/zootaxa.119.1.1)

[76] Clarkston BE, Saunders GW. 2010 A comparison of two DNA barcode markers for species discrimination in the red algal family Kallymeniaceae (Gigartinales, Florideophyceae), with a description of *Euthora timburtonii* sp. nov. Botany **88**, 119-131. (10.1139/B09-101)

[77] Fernandez-Triana J. 2018 Ten unique and charismatic new species of Microgastrinae wasps (Hymenoptera, Braconidae) from North America. ZooKeys **730**, 123-150. (10.3897/zookeys.730.22869) PMC5799786 29416399

[78] Marshall AJ, Blanchon DJ, Lücking R, de Lange TJP, de Lange PJ. 2020 A new *Ocellularia* (lichenized Ascomycota: Graphidaceae) from New Zealand indicates small-scale differentiation of an Australasian species complex. N. Z. J. Bot. **58**, 223-235. (10.1080/0028825X.2019.1701504)

[79] Mebert K, González-Pinzón M, Miranda M, Griffith E, Vesely M, Schmid PL, Batista A. 2022 A new rainfrog of the genus *Pristimantis* (Anura, Brachycephaloidea) from central and eastern Panama. ZooKeys **1081**, 1-34. (10.3897/zookeys.1081.63009) 35087294 PMC8763812

[80] Kohler R. 1995 A new species of the fossil turtle *Psephophorus* (Order Testudines) from the Eocene of the South Island, New Zealand. J. R. Soc. N. Z. **25**, 371-384. (10.1080/03014223.1995.9517495)

[81] Blake K, Anderson S, Gleave A, Veríssimo D. In press. Impact on species' online attention when named after celebrities. Conserv. Biol. (10.1111/cobi.14184) 37700661

[82] Karaffa PT, Draheim MM, Parsons ECM. 2012 What's in a name? Do species' names impact student support for conservation? Hum. Dimens. Wildl. **17**, 308-310. (10.1080/10871209.2012.676708)

[83] Díaz-Restrepo A, Balcombe K, Fraser I, Smith RJ, Veríssimo D. 2022 Testing branding techniques on species common names to improve their fundraising profile for conservation. Anim. Conserv. **25**, 27-37. (10.1111/acv.12715)

[84] Blades B. 2020 What's in a name? An evidence-based approach to understanding the implications of vernacular name on conservation of the painted dog (*Lycaon pictus*). Lang. Ecol*.* **1**, 1-27.

[85] Halverson K, Heard SB, Nason JD, Stireman JO. 2008 Differential attack on diploid, tetraploid, and hexaploid *Solidago altissima* L. by five insect gallmakers. Oecologia **154**, 755-761. (10.1007/s00442-007-0863-3) 17924147

[86] Heard SB, Stireman JO, Nason JD, Cox GH, Kolacz CR, Brown JM. 2006 On the elusiveness of enemy-free space: spatial, temporal, and host-plant-related variation in parasitoid attack rates on three gallmakers of goldenrods. Oecologia **150**, 421-434. (10.1007/s00442-006-0529-6) 16944244

[87] Heard SB, Cox GH. 2009 Plant module size and attack by the goldenrod spindle-gall moth. Can. Entomol. **41**, 404-416.

[88] Heard SB, Kitts EK. 2012 Impact of attack by *Gnorimoschema* gallmakers on their ancestral and novel *Solidago* hosts. Evol. Ecol. **26**, 879-892. (10.1007/s10682-011-9545-z)

[89] Chambers J, Sparks N, Sydney N, Livingstone PG, Cookson AR, Whitworth DE. 2020 Comparative genomics and pan-genomics of the Myxococcaceae, including a description of five novel species: *Myxococcus eversor* sp. nov., *Myxococcus llanfairpwllgwyngyllgogerychwyrndrobwllllantysiliogogogochensis* sp. nov., *Myxococcus vastator* sp. nov., *Pyxidicoccus caerfyrddinensis* sp. nov., and *Pyxidicoccus trucidator* sp. nov. Genome Biol. Evol. **12**, 2289-2302. (10.1093/gbe/evaa212) 33022031 PMC7846144

[90] Knapp S, Vorontsova MS, Turland NJ. 2020 Indigenous species names in algae, fungi and plants: a comment on Gillman & Wright (2020). Taxon **69**, 1409-1410. (10.1002/tax.12411)

[91] Westoby M. 2002 Choosing species to study. Trends Ecol. Evol. **17**, 587. (10.1016/S0169-5347(02)02634-4)

[92] Dietrich MR, Ankeny RA, Crowe N, Green S, Leonelli S. 2020 How to choose your research organism. Stud. Hist. Philos. Sci. Part C Stud. Hist. Philos. Biol. Biomed. Sci. **80**, 101227. (10.1016/j.shpsc.2019.101227) 31883711

[93] Heard SB, Mlynarek JJ. 2023 Naming the menagerie: creativity, culture, and consequences in the formation of scientific names. Figshare. (10.6084/m9.figshare.c.6890518) PMC10618856 37909078

